# Family structure and multisite musculoskeletal pain in adolescence: a Northern Finland Birth Cohort 1986 study

**DOI:** 10.1186/s12891-023-06294-0

**Published:** 2023-03-11

**Authors:** Eveliina Heikkala, Petteri Oura, Jaro Karppinen, Annie Herbert, Heidi Varis, Maria Hagnäs, Ilona Mikkola, Markus Paananen

**Affiliations:** 1 Research Unit of Population Health, University of Oulu, PO Box 8000, 90014 Oulu, Finland; 2 Medical Research Center Oulu, University of Oulu and Oulu University Hospital, 90014 Oulu, Finland; 3 Wellbeing Services, County of Lapland, 96400 Rovaniemi, Finland; 4 Research Unit of Health Sciences and Technology, University of Oulu, Oulu, Finland; 5 Rehabilitation Services of South Karelia Social and Health Care District, 53130 Lappeenranta, Finland; 6 Department of Population Health Sciences, University of Bristol, Bristol, UK; 7 MRC Integrative Epidemiology Unit, University of Bristol, Oakfield House, Oakfield Grove, BS8 2BN Bristol, UK; 8 Primary Health Care Services, City of Espoo, 02070 Espoo, Finland

**Keywords:** Family structure, Multisite musculoskeletal pain, Adolescent, Birth cohort study

## Abstract

**Background:**

Family structure is suggested to be associated with adolescent pain, but evidence on its association with multisite MS pain is sparse. The purpose of this cross-sectional study was to investigate the potential associations between family structure (‘single-parent family’, ‘reconstructed family’, and ‘two-parent family’) and multisite musculoskeletal (MS) pain in adolescence.

**Methods:**

The dataset was based on the 16-year-old Northern Finland Birth Cohort 1986 adolescents with available data on family structure, multisite MS pain, and a potential confounder (n = 5,878). The associations between family structure and multisite MS pain were analyzed with binomial logistic regression and modelled as unadjusted, as the evaluated potential confounder, mother’s educational level, did not meet the criteria for a confounder.

**Results:**

Overall, 13% of the adolescents had a ‘single-parent family’ and 8% a ‘reconstructed family’. Adolescents living in a single-parent family had 36% higher odds of multisite MS pain compared to adolescents from two-parent families (the reference) (Odds Ratio [OR]: 1.36, 95% Confidence Interval [CI]: 1.17 to 1.59). Belonging to a ‘reconstructed family’ was associated with 39% higher odds of multisite MS pain (OR 1.39, 1.14 to 1.69).

**Conclusion:**

Family structure may have a role in adolescent multisite MS pain. Future research is needed on causality between family structure and multisite MS pain, to establish if there is a need for targeted support.

**Supplementary Information:**

The online version contains supplementary material available at 10.1186/s12891-023-06294-0.

## Background

Higher divorce rates and an increased prevalence of single-parent families have diversified the family environment in which children and adolescents grow up today [1–3]. For instance, in 2016, 27% of children and adolescents in the United States lived with a single parent, while 56 years earlier, the corresponding prevalence was only 9% [3]. Expectedly, the field has received a lot of research attention, and a growing body of evidence implies that living in two-parent families in comparison to single-parent families is more favorable to psychological well-being and health in general [4–6]. However, some health outcomes have not been sufficiently explored in relation to family structure. One such example is musculoskeletal (MS) pain. Adverse childhood experiences, including parental separation, have been associated with chronic pain in childhood/adolescence and these associations have been suggested to be attributable to biological, psychological, and social elements related to greater risk for pain [7]. This sets up the framework for additional research on family structure and pain.

MS pain is experienced in the muscles, tendons, ligaments and/or bones in the MS system (e.g. neck and back) and is a common symptom among children and adolescents, with an estimated prevalence of up to 40% before turning 18 [8]. Usually MS pain is non-specific, self-healing, and causes no long-term disability, but quite often it can become chronic and disabling, impairing e.g. participation in sports or school [8–10]. Existing literature has suggested that multisite MS pain, pain which occurs in several locations in the MS system at a given time, is especially disabling [11, 12] and relates to lowered health-related quality of life more strongly than single-site pain [12, 13]. Multisite MS pain has a substantial tendency to persist into adulthood [14, 15] and has a negative effect on labor market engagement and social inclusion among adults [16–18].

A wide spectrum of sociodemographic and health-related determinants of adolescents’ multisite MS pain have been identified in the existing literature [19]. To date, however, the role of family structure in adolescent pain is underexplored and unclear, with contradictory findings being published [20–23]. We are especially lacking studies on adolescent multisite MS pain and family structure even though they might also be related [24]. Moreover, adolescents living in reconstructed families (that is, those involving a step-parent or a parent’s partner), and adolescents living with a single parent, have often been combined in previous analyses, although it may be that the odds of pain vary between these family structures, as suggested by some studies in terms of other health problems (e.g., weight status [25]) and health-related quality of life [26]. Identifying high-risk groups and determinants of multisite MS pain can guide public health efforts, to reduce both suffering for the individuals as well as downstream socio-economic effects.

In order to understand the relevance of family structure in multisite MS pain, the present study employed the Northern Finland Birth Cohort 1986 (NFBC1986) to study the associations between family structure (divided into ‘single-parent family’, ‘reconstructed family’, and ‘two-parent family’) and multisite MS pain, both measured when the adolescents were 16 years. We hypothesized that adolescents living in a two-parent family structure would be the lowest risk group for multisite MS pain, but had no prior hypotheses about who would be the highest risk group between a single parent and reconstructed family.

## Methods

### Study aim

This study aimed to examine the associations between family structure (divided into ‘single-parent family’, ‘reconstructed family’, and ‘two-parent family’) and multisite MS pain in adolescence.

### Study design and study population

NFBC1986 is an established mother-child birth cohort, which has been followed regularly since the offspring’s birth between 1985 and 1986 in the northernmost provinces of Finland (Oulu, Lapland; n = 9479) [27]. The detailed description of the study design and NFBC1986 is provided elsewhere [28]. The current study concentrates on the 16-year data collection point, during which questionnaires were posted to the adolescents and their caregivers.

**Fig. 1:**
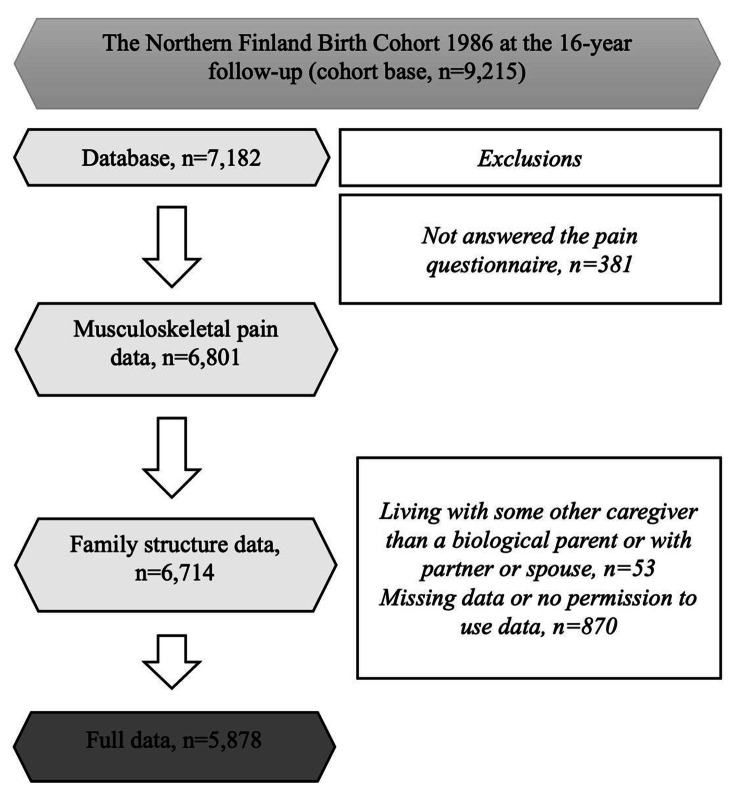
Flowchart of the sample selection

At 16 years, 7,182 (78% of the cohort base) of the adolescents and 6,866 (75%) of their caregivers returned the filled in questionnaires. After exclusions (Fig. 1), the full data on family structure, multisite MS pain, and a potential confounder were available for 5,878 adolescents (64% of the cohort base) which comprised the study sample for the analyses. All the adolescents and their caregivers gave their written consent to use data in the research. The study protocol was approved by the Northern Ostrobothnia Hospital District Ethical Committee 108/2017 (15.1.2018).

### Multisite musculoskeletal pain

Multisite MS pain was defined as pain in three or more locations of the MS system in line with the previous finding that the health-related quality of life is reduced especially among those adolescents reporting three or more pain locations [13]. The question “Have you had any aches or pains during the last six months in the following areas of your body?” asked participants to evaluate their MS pain in (1) neck or occipital area, (2) shoulders, (3) low back, (4) elbows, (5) wrists, (6) knees, and (7) ankle–foot area, followed by a drawn mannequin indicating these locations. The adolescents were given three response options in each potential pain location: (1) No, (2) Yes, but I have not consulted a physician, physiotherapist, nurse or other health professional because of my pain, or (3) Yes, and I have consulted a physician, physiotherapist, nurse or other health professional because of my pain. For each pain location, the last two categories were combined to make a binary variable: no pain vs. any pain. Adolescents not responding to some of the pain locations were regarded as having no pain in these locations, including 3% adolescents who had at least partly filled in the pain questionnaire. The 4% of adolescents who had not filled in the MS pain questionnaire at all were excluded. Then, a multisite MS pain score variable was created by summing all endorsed pain locations (0–7 locations), and dichotomizing the sum as 0–2 locations (‘no multisite MS pain’) and three or more locations (‘multisite MS pain’).

### Family structure

Family structure was evaluated by enquiring about the living conditions at the age of 16 years from the adolescents and their caregivers. The question “Who do you mainly live with?” was included in the members’ questionnaire with response options of (1) mother and father, (2) mother and stepfather, (3) father and stepmother, (4) only mother, (5) only father, (6) some other caregiver (e.g. foster parent), and (7) own partner or spouse. In the survey sent to the caregivers, caregivers were asked to describe their marital status and living arrangements as follows: “Which one of the following describes best the marital status of the child’s principal caregiver?” and “Do the child’s biological parents live together?” The response options for the former question were (1) married or cohabiting with a child’s own mother or father, (2) divorced and single parent, (3) divorced and shared custody, (4) divorced and reconstructed family, (5) single, and (6) widow/widower; and for the latter question: (1) yes, (2) no, because they have divorced, (3) no, because they have not lived together at all, and (4) no, because other parent has died. On the basis of the aforementioned questions, the following categories were formulated for analysis: ‘two-parent family’, ‘reconstructed family’, and ‘single-parent family’. Caregiver’s response was prioritized; if not available, adolescent’s response was used. The ‘two-parent family’ category was used as the reference in analysis.

### Potential confounder

The primary criteria for a potential confounder was a hypothesized association with both the exposure (family structure) and the outcome (multisite MS pain) in a way that the variable cannot be considered as a mediator or collider factor. Mother’s educational level (a proxy for socioeconomic status) was considered as a potential confounder, which was recorded when the adolescent was age 16. Mother’s educational level was categorized as follows: (1) basic education (a maximum of nine years), (2) upper secondary education (10–12 years), (3) tertiary education (13 or more years), and (4) other or degree not finished [29]. Before inclusion in the final analyses, the statistical significance of the hypothesized associations was tested (please see Statistical analysis for further information).

### Descriptive variables

Sex, a number of adolescents’ health behaviors (physical activity level, smoking status, and average sleeping duration) and psychosocial problems (emotional and behavioral problems), recorded also at 16 years, were used to characterize the study sample. These variables were not considered as potential confounders in analyses as they did not *a priori* fulfil the criteria for a potential confounder.

Adolescent’s sex was captured from the birth record. The level of brisk physical activity (physical activity causing at least some sweating and shortness of breath) outside school hours per week was divided into three categories: one hour or less (inactive), 2–3 h (moderate active), and more than three hours (active). Adolescents were distributed into three categories according to their smoking behavior: non-smokers, one pack-year or less by the age of 16 years, and over one pack-year, where one pack-year is equivalent to 15 cigarettes smoked per day for a year [19]. Reported average sleeping duration was dichotomized as follows: recommended (8–10 h) and non-recommended (under 8 h or over 10 h) in accordance with the current guidelines [30]. Adolescents’ emotional and behavioral problems were captured using a valid and reliable Youth Self-Report (YSR) questionnaire [31, 32] and evaluated as dichotomized variables (normal range vs. problem range). The precise description of the questionnaire and procedure of the formulation of the emotional and behavioral problems variables have been presented elsewhere [32].

### Statistical analysis

Analyses were performed using the statistical program SPSS, version 27.0 (IBM, Armonk, NY, USA). We first presented descriptive statistics (numbers and proportions) of multisite MS pain, mother’s education level, and other descriptive variables by family structure categories, and for the total sample. The associations between family structure and multisite MS pain were explored through binomial logistic regression analysis, presented as odd ratios (ORs) and their 95% confidence intervals (CIs). The univariate association of the potential confounder with both the family structure and multisite MS pain were tested using multinomial logistic regression analysis. Although there was an association between mother’s educational level and family structure (e.g. [multinomial] odds ratios of ‘reconstructed family’ vs. ‘two-parent family’ for upper secondary education vs. tertiary education = 1.51 [95% CI 1.08–2.09]), there was little evidence of an association between mother’s education level and multisite MS pain (Supplement 1). As such, the associations between family structure and multisite MS pain were modelled as unadjusted.

To explore the potential modifying role of sex (i.e. any need to stratify analyses by sex), we modelled the relationship between family structure and multisite MS pain, including an interaction term (sex*family structure) as an independent variable, but found very weak evidence of an interaction (p = 0.906). Given that the outcome of multisite MS pain was fairly common (34%), we also present (unadjusted) risk ratios, estimated from log-binomial models.

## Results

Of 5,878 adolescents in the study sample, 8% of them lived in a ‘reconstructed family’ and 13% in a ‘single-parent family’ (the remaining 79% in a two-parent family; Table 1). A higher percentage of the adolescents in these family categories reported multisite MS pain (reconstructed: 40%, single-parent: 40%, two-parent: 33%). They were also more likely to have mothers with non-tertiary education (tertiary education: 9% and 12% vs. 13%, respectively), and generally to be more likely engaged in unhealthy behaviors and have emotional and behavioral problems, compared with adolescents who lived in the ‘two-parent family’ (e.g. 41% and 38% physically inactive vs. 34%; 20% and 18% had emotional problems vs. 15%).

**Table 1:** Characteristics of the family structure categories at 16 years, % (n)

	‘Single-parent family’ (n = 780)	‘Reconstructed family’ (n = 457)	‘Two-parent family’ (n = 4,641)	All (n = 5,878)	
Variables					
Sex					
Boys	49 (379)	43 (197)	49 (2,272)	48 (2,848)	
Girls	51 (401)	57 (260)	51 (2,369)	52 (3,030)	
Mother’s educational level					
Basic education	9 (66)	8 (37)	8 (379)	8 (482)	
Upper secondary education	67 (524)	71 (325)	68 (3,126)	68 (3,975)	
Tertiary education	12 (97)	9 (43)	13 (623)	13 (763)	
Other or unfinished	12 (93)	11 (52)	11 (513)	11 (658)	
Physical activity level					
Inactive	38 (292)	41 (186)	34 (1,584)	35 (2,062)	
Moderate active	27 (212)	25 (118)	27 (1,241)	27 (1,571)	
Active	34 (266)	33 (150)	38 (1,779)	37 (2,195)	
Missing	1 (10)	1 (3)	1 (37)	1 (50)	
Smoking status					
Non-smoker	73 (565)	71 (322)	86 (3,983)	83 (4,870)	
One pack-year or less	14 (112)	14 (65)	9 (418)	10 (595)	
Over one pack-year	13 (103)	15 (70)	5 (240)	7 (413)	
Average sleeping duration					
Recommended	71 (552)	71 (325)	79 (3,656)	77 (4,533)	
Non-recommended	28 (218)	29 (131)	20 (949)	22 (1,298)	
Missing	1 (10)	0 (1)	1 (36)	1 (47)	
Emotional problems					
Problem range	18 (142)	20 (90)	15 (671)	15 (903)	
Normal range	82 (638)	80 (367)	85 (3,970)	85 (4,975)	
Behavioral problems					
Problem range	26 (200)	31 (140)	19 (874)	21 (1,214)	
Normal range	74 (580)	69 (317)	81 (3,767)	79 (4,664)	
Multisite MS pain					
No	60 (469)	60 (273)	67 (3,122)	66 (3,864)	
Yes	40 (311)	40 (184)	33 (1,519)	34 (2,014)	

Pack-year = 15 cigarettes smoked per day for a year.

MS = musculoskeletal.

Table 2 represents the logistic models for the associations between family structure and multisite MS pain. Adolescents in the ‘single-parent family’ and ‘reconstructed family’ categories had 36% and 39% higher odds of multisite MS pain, respectively, when compared to the adolescents of the ‘two-parent family’ structure (single-parent: OR 1.36, 95% CI 1.17–1.59; reconstructed: OR 1.39, 95% CI 1.14–1.69, respectively). Though risk ratios were slightly smaller than odds ratios, patterns were similar (positive with the largest being for ‘reconstructed family’).

**Table 2:** Unadjusted associations between family structure and multisite musculoskeletal (MS) pain at 16 years (n = 5,878), presented as odds ratios, risk ratios, and 95% confidence intervals

	Multisite musculoskeletal pain	
	Odds ratios	Risk ratios
‘Single-parent family’	**1.36** (1.17–1.59)	**1.22** (1.11–1.34)
‘Reconstructed family’	**1.39** (1.14–1.69)	**1.23** (1.09–1.39)
‘Two-parent family’	Ref.	Ref.

Statistically significant values at the 5% level are bolded.

## Discussion

In this study on 16-year-old Northern Finns, we aimed to explore the associations between family structure and multisite MS pain. Adolescents living in a ‘single-parent family’ had 36% higher odds of multisite MS pain, compared to adolescents from two-parent families (95% CI: 17–59%). Similarly, belonging to a ‘reconstructed family’ was associated with 39% higher odds of multisite MS pain (95% CI 14–69%).

Of 5,878 adolescents, 79% lived in two-parent families, 13% in single-parent families, and 8% in reconstructed families. In general, this distribution corresponds quite well with the reports based on national registers from year 2018 [33] and those of other Nordic countries in which typically ca. 70% of adolescents live in two-parent families [34, 35], given that our data were collected over 20 years ago. In addition, the estimated family structure categories were not restricted to traditional family structures due to the used questions and their response options, thus potentially characterizing a diversity of family structures, such as two-parent families with same sex parents, even though being divided into three categories. Adolescents living with two parents seemed to have adopted a more favorable lifestyle and express fewer psychosocial symptoms in relation to their counterparts in other family structures, which is a recognized phenomenon in the literature [4, 36–38]. Prevalence of multisite MS pain was also the lowest among the two-parent family adolescents, but nevertheless high at 33%. Estimates of the overall prevalence of adolescent multisite MS pain have varied between 22% and 37% in the existing literature when using three pain sites as the cut-off point for multisite MS pain [14, 16, 39], being in concordance with our findings. Overall, these considerations and the large population-based sample are likely to increase the generalizability of the current results not only in a geographical manner but also in terms of present time.

Adolescents who lived in the ‘single-parent family’ had 36% higher odds of multisite MS pain and adolescents living in the ‘reconstructed family’ had 39% higher odds of multisite MS pain during adolescence, compared with counterparts who lived with two biological parents. These estimates remained similar regardless of adjustments. In a previous one-year follow-up study, parental divorce was associated with frequent multisite pain in adolescence [22], and some studies have reported associations between childhood maternal death [40] and parental divorce [41] with widespread pain in adulthood. Even though the outcomes of these studies measure family structure differently, do not necessarily fully correspond to multisite MS pain, and report on outcomes at different follow-up times compared to our cross-sectional study, our findings indicate similar relationships. Our study is among the first to show that not only living with a single-parent after family environment-related stressors encompassing parental death or separation but also adjustment to rearranged family environmental including one or more new family members are associated with adolescents’ multisite MS pain. Still, it is essential to note that not all single-parent families or reconstructed families are identical, i.e. they may have different levels of support network including grandparents and friends or the family environment may be otherwise favorable or satisfactory to an adolescent, which may be of importance in terms of how family structure influences (MS) health on an individual level.

The underlying mechanisms for the observation that adolescents from the single-parent families and reconstructed families have higher odds of multisite MS pain are likely to be manifold, potentially including biological, psychological, and social elements [7]. Adverse childhood experiences, including parental separation or death, may lead to anxiety and mood disorders and/or unhealthy behaviors, e.g. smoking [36], which, in turn, may precede the development of a higher number of painful conditions [42, 43]. It has also been proposed that chronic stress in general, predisposed by adverse childhood experiences, induce endocrine changes that may expose to pain [44]. Moreover, it may be that caregivers themselves in the single-parent and reconstructed families have more pain, influencing offspring’s pain reports [45]. This suggestion is supported by a study by Hoftun et al., [35], where the odds of chronic multisite pain were found to be slightly higher among adolescents whose mothers suffered from chronic pain if the adolescent lived with the mother alone or in a reconstructed family, in relation to adolescent who lived with both parents.

Unfortunately use of pain medication for MS pain was not available in the data. It is possible that the way in which pain medication is used to treat multisite MS pain differs between family structure types. For example, single parent families may have fewer resources than two-parent or reconstructed families [46] and therefore may have been less likely to treat pain, before their multisite MS pain status was recorded (i.e. resulting in differential classification of the outcome). In this example, the effect of single-parent family on multisite MS pain would be under-estimated.

A representative birth cohort sample of Northern Finnish adolescents with large population base and relatively high response rate comprise definite strengths of the present study. This study is also among the first adolescent studies on multisite MS pain and family structure. However, there are a few limitations that need to be addressed. Firstly, MS pain questions assessed only pain that occurred during the previous six months without inquiry of pain frequency, severity, disability or etiology. Therefore, the outcome of multisite MS pain is a crude measure in this study, and does not pick up on subtle variations in pain levels or the full context of suffering from MS pain – this would mean that models would be less likely to report associations that truly exist – nevertheless there is evidence of a positive association in the study. Secondly, as for family structure, we had no sufficient data on the point of time when parental separation or death had occurred and when the reconstructed family had been built up. Though our study captured family structure and multisite MS pain at the same time-point, it is likely that any effect of family structure on pain is a long-term cumulative one. However, we cannot disentangle when exactly an individual may have reached the threshold for multisite MS pain in relation to when family structure was established. Thirdly, there may be other variables present prior to age 16 that confound the relationship between family structure and MS pain, e.g. factors such as parental mental or physical health, which were not available in the data. Inclusion of such variables in analyses would likely attenuate effect estimates reported in the current study. Finally, considering the relatively low number of parental deaths reported in this dataset (2%), we were unable to estimate potential differences in the ‘single-parent’ group, between adolescents who were affected by parental death or separation. Finally, being a questionnaire-based study, existence of recall bias cannot be ruled out.

## Conclusion

This study on a large birth cohort showed that adolescents living in a single-parent or reconstructed family have higher odds of multisite MS pain than adolescents living in two-parent families. Our findings extend the literature by indicating that family structure should potentially be considered when evaluating the risk of adolescent multisite MS pain in the clinical context, and that there may be a need for targeted support among adolescents living with a single parent or in a reconstructed family. As these adolescents are likely to be vulnerable to other health complaints as well [4–6], public-level preventative actions may be required to improve health and well-being in adulthood. In future, additional research is needed to establish causality between family structure and multisite MS pain. Moreover, potential underlying mechanisms for the association between single-parent and reconstructed families, and multisite MS pain are to be investigated in more detail.

## Electronic supplementary material

Below is the link to the electronic supplementary material.

Supplementary Material 1

## Data Availability

NFBC data are available from the University of Oulu, Infrastructure for Population Studies. Permission to use the data can be applied for research purposes via an electronic material request portal. In the use of data, we followed the EU general data protection regulation (679/2016) and the Finnish Data Protection Act. The use of personal data is based on a cohort participant’s written informed consent in their latest follow-up study, which may cause limitations to its use. Please contact the NFBC project centre (NFBCprojectcenter@oulu.fi) and visit the cohort website (www.oulu.fi/nfbc) for more information.

## Abbreviations

CI: confidence interval
MS: musculoskeletal
NFBC1986: Northern Finland Birth Cohort 1986
OR: odds ratio
YSR: Youth Self-Report
