# Supplementary material for: Family structure and multisite musculoskeletal pain in adolescence: a Northern Finland Birth Cohort 1986 study
Source: BMC Musculoskelet Disord. 2023 Mar 11;24:185. doi: 10.1186/s12891-023-06294-0 (PMC10007855; doi:10.1186/s12891-023-06294-0)
Supplement: Supplementary file 1 — Supplementary Material 1 [file 12891_2023_6294_MOESM1_ESM.docx]

| **Supplement 1. Univariate associations of the potential confounder with both the family structure and multisite musculoskeletal (MS) pain at 16 years, presented as odds ratios (ORs) and 95% confidence intervals (CIs).** | | | | | | |
| --- | --- | --- | --- | --- | --- | --- |
|  | Family structure | | | | | |
|  | ‘Single-parent family’ (n=780) | | ‘Reconstructed family’ (n=457) | | | ’Two-parent family’ (n=4,641) |
| Potential confounder |  | |  | | |  |
| Mother’s educational level |  | |  | | |  |
| Basic education | 1.12 (0.80–1.57) | | 1.41 (0.89–2.24) | | | Ref. |
| Upper secondary education | 1.08 (0.85–1.36) | | **1.51** (1.08–2.09) | | | Ref. |
| Tertiary education | Ref. | | Ref. | | |  |
| Other or unfinished | 1.16 (0.86–1.58) | | 1.47 (0.96–2.24) | | | Ref. |
|  |  |  | | |  | |
|  | Multisite MS pain | | | | | |
|  | Yes (n=2,014) | | | No (n=3,864) | | |
| Mother’s educational level |  | | |  | | |
| Basic education | 1.00 (0.79–1.28) | | | Ref. | | |
| Upper secondary education | 1.03 (0.87–1.21) | | | Ref. | | |
| Tertiary education | Ref. | | |  | | |
| Other or unfinished | 0.98 (0.78–1.22) | | | Ref. | | |

Statistically significant values at the 5% level are bolded.

Odds ratios estimated from multinomial logistic regression.
